# Therapeutic Benefit of *Dillenia indica* in Diabetes and Its Associated Complications

**DOI:** 10.1155/2019/4632491

**Published:** 2019-11-23

**Authors:** Parul Kamboj, Narayan C. Talukdar, Sanjay K. Banerjee

**Affiliations:** ^1^Translational Health Science and Technology Institute (THSTI), -121001, Faridabad, India; ^2^Institute of Advanced Study in Science and Technology, -781035, Guwahati, Assam, India

## Abstract

Diabetes, a metabolic disorder characterized by elevated fasting blood glucose levels, affects nearly 8% of the world population and was predicted that it would be the top seven leading cause of death in the next ten years. The incidence of diabetes and its morbidity are increasing rapidly in developing countries due to lifestyle change and intake of high-calorie diet occurring with urbanization. Medicinal plants and their products have been proven to be effective, less expensive, and safe for the treatment and prevention of diabetes. Although several medicinal plants known for the antidiabetic property are reported in the ancient medical textbook, there is always a scope to identify and validate less explored medicinal plants that are still practiced regularly by local and tribal people since ancient times. Here, in the present article, we would like to review a less explored medicinal plant, *Dillenia indica*, which has promising effects in treating diabetes and other diabetic-associated complications. In spite of its wide use in the Northeast region of India as traditional medicine, there is only one clinical study where the antidiabetic potential of the fruit powder has been shown. Further well-designed animal and human studies are needed to confirm the role of *Dillenia indica* in diabetes and its associated complications.

## 1. Introduction

Diabetes mellitus (DM) is a chronic metabolic complication associated with the incidents of glucose intolerance and hyperglycemia. Chronic hyperglycemia results in poor insulin deficiency and defects in the action of insulin with a growing loss of beta-cell function or both and it is concomitant with long-term damage and impairment in the function of various tissues and organs like the eyes, heart, blood vessels, kidneys, and nerves [1]. DM is linked with pancreas and inslin secretion, it may affect when the pancreas does not produce (type 1 diabetes) sufficient amount of insulin (a hormone, which regulates the blood sugar level) or when the body does not utilize (type 2 diabetes) enough amount of insulin produced by the pancreas. Diabetes is a chronic and complex disease involving multiple morbidities that requires the attention of multiple health care providers or facilities [2]. It is one of the world's major health problems and predicted that it would be the top seven leading cause of death in the next ten years. A total of 422 million adults have been reported worldwide with diabetes in 2014 and there is a huge difference compared to 108 million in 1980. The prevalence of diabetes in the adult population has become nearly doubled since 1980, rising from 4.7% to 8.5% [3]. In 2012, 1.5 million deaths were reported from diabetes [4]. The number of diabetic cases and its prevalence have been increasing widely from the past few years. Elevated blood glucose levels caused an additional 2.2 million death by increasing the risk of cardiovascular disease and associated complications. Diabetes and its associated complications increase the overall risk of a fatal death. The possible complications include kidney failure, liver dysfunction, heart attack, stroke, vision loss, and nerve damage. Thus, diabetes care is complex and requires many issues, beyond glycemic control, to be addressed [5]. Diabetes and its associated complications bring about extensive economic forfeiture to people with diabetes and their families, and to health systems and national economies through direct medical costs and loss of work and wages [4]. Although there are several drugs which can control high blood glucose level in diabetes, none of them are suitable to reduce organ damage associated with diabetes. Again, some of the drugs show severe hypoglycemia and cause further complications. Therefore, there is an urgent need to look for an alternative therapy, especially natural products which could be a useful for the very early stage of diabetes, like prediabetes and attenuate the disease progression and its pathophysiology.

Herbal plants are the richest source of drugs in India from prehistoric times. Herbal drugs are beneficial for mankind in treating various diseases. Plants play a very important role in the life of various animals and humans and act as the backbone of all forms of life on Earth [6]. There are about 800 medicinal plants that have been reported worldwide for their antidiabetic activity and used as herbal home remedies or as the remedy of grandmother [7]. Although more than 400 species with hypoglycemic activity were reported previously, investigation of new antidiabetic drugs from natural plants is still striking. Recently, explored several medicinal plants contain various substances with unique beneficial effects on diabetes and its associated complications. Most of the plants contain various active constituents like alkaloids, terpenoids, flavonoids, glycosides, and polyphenols, which often together have an antidiabetic effect [8]. Further, investigation and validation of traditional knowledge should be encouraged for finding a better therapy to cure diabetes. Here, in the present review, we are going to discuss the use of *Dillenia indica*, an Indian traditional plant, in diabetes and its potential for antidiabetic therapy in the future.

## 2. *Dillenia indica*: Description and Its Traditional Use

**Figure 1:**
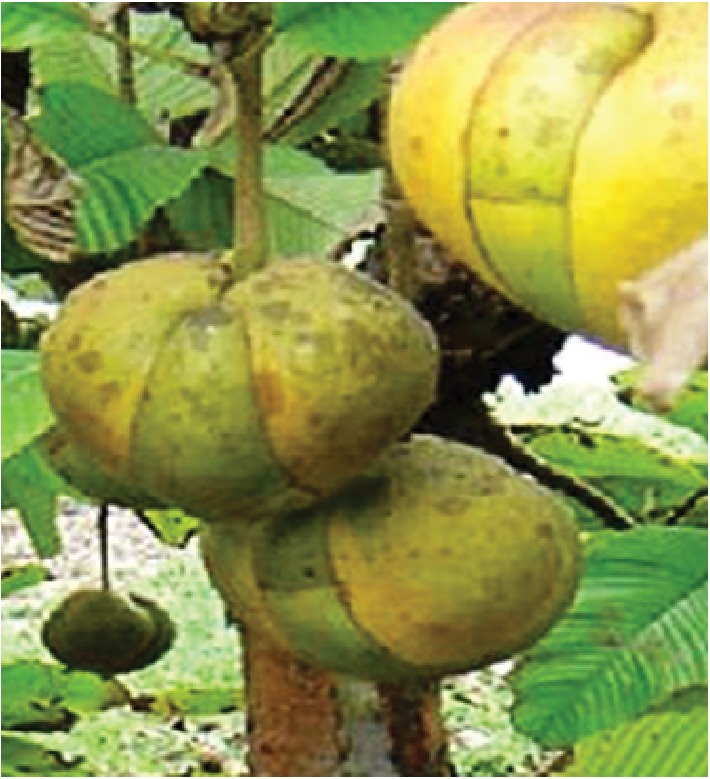
*Dillenia indica* plant and fruit adopted from a review [9].

*Dillenia indica* (Figure 1) is commonly known as elephant apple. This plant has several other names as represented in Table 1. This plant is classified scientifically in various subclasses, division, and family according to the botanical scheme as represented in Table 2. It belongs to the family *Dilleniaecae*. It is the most favorable herb of the Assamese (people from state Assam, India) cuisine. The pulp of the fruit is applied on the scalp to cure dandruff and hair fall, and the sepals of this plant have been used from ancient times to treat stomach disorders [9]. It is one of the herbs that was widely used in the tribal areas of Northeast India including Assam [10]. The plant can also be found in other countries like Bhutan, China, Sri Lanka, Indonesia, Malaysia, Myanmar, Nepal, the Philippines, Thailand, and Vietnam [11, 12]. Medicinal uses of this plant were described in different ancient texts like Yajurveda, Charka Samhita, Sushruta Samhita, Rajanighantu, Matsya Purana, Agni Purana, and Flora of China [13, 14].

It is one of the evergreen plants found in wild areas. It is a 15-meter tall large shrub or small- to medium-sized semideciduous having branches on the tree. It is grown in moist areas of India. Leaves of this plant are fascicled at the end of the branches, lanceolate, 20-30 cm long, and with a sharp notch. The plant has a white-colored large flower, which is 15 cm in diameter, solitary, and end to end branched. It has a hard and large fruit 3-5 inch diameter [9, 15]. The literature review of this plant has shown various medicinal properties in the form of fruit, leaves, bark, or other parts of the plant. The plant possesses various medicinal properties like cancer, astringent, diarrhea, and laxative [16, 17]. It is used to treat fever and acts as a cooling agent to relieve body heat [18, 19]. It also possess several other activities like antimicrobial [20, 21], antioxidant [22, 23], analgesic [24], anti-inflammatory, and dysentery [17, 25]. Beside all these activities, various parts of this plant are found to be beneficial in treating diabetes [8, 26] and its associated complications like hyperlipidemia [15, 27], diabetic nephropathy, and neuropathy as represented in Table 3 [28, 29]. In the present review, an attempt was made to focus on *Dillenia indica* for its antidiabetic use along with its complication treatment. Scientific classification and other names of this plant in different languages are given in Table 1 and Table 2, respectively.

## 3. Therapeutic Potential of *Dillenia indica* in Diabetes: Preclinical Study

### 3.1. *Dillenia indica* in Diabetes

Various studies have shown the medicinal value of *Dillenia indica*, which is commonly used by the local people of Northeast region of India. Data from various studies and folklore evidence stated that *Dillenia indica* might be beneficial in the management of diabetic mellitus. A study on Wistar rats showed significant antidiabetic activity at a dose of 250 and 500 mg/kg body weight in streptozotocin- (STZ-) induced diabetic model [15]. Enhancement of serum insulin level was reported after treatment with methanolic leaf extract of *Dillenia indica* [15, 27, 30]. The extract treatment also showed an enhanced body weight of diabetic rats as compared to the diabetic control group [27]. In another study, alcoholic extract of *Dillenia indica* (DAE) was subjected to column chromatography for the isolation of a new compound, chromane [3,5,7-trihydroxy-2-(4-hydroxybenzyl)-chroman-4-one]. In this study, diabetes was induced in rats by the administration of STZ at a dose of 50 mg/kg body weight. To find the efficacy, the alcoholic extract of *Dillenia indica* (DAE) was administered to diabetic rats at 100, 200, and 400 mg/kg dose, while the isolated compound chromane was administered to diabetic rats at 5 and 10 mg/kg dose orally. Data showed that DAE and chromane significantly decrease elevated fasting blood glucose, lipid levels, and oxidative stress. The study concluded that *Dillenia indica* and its isolated compound proved to have a strong antidiabetic effect and could be beneficial in the management of diabetes [26].

### 3.2. *Dillenia indica* in Diabetes-Associated Dyslipidemia

Dyslipidemia is the major leading factor causing atherosclerosis. Dyslipidemia contributes to increasing plasma lipids including triglycerides, cholesterol, cholesterol esters, and phospholipids and plasma lipoproteins, i.e., very low-density lipoprotein and low-density lipoprotein and reduced high-density lipoprotein levels [31, 32]. Kumar et al. reported the hypolipidemic activity of *Dillenia indica* (methanolic leaves extract) in alloxan-induced diabetic rats at a dose of 200 and 500 mg/kg body weight. Diabetes were induced in rats by a single intraperitoneal injection of alloxan monohydrate at a dose of 150 mg/kg body weight. Twenty-one days treatment of *Dillenia indica* extract found to be beneficial in improving the total lipid levels, body weight, liver, and kidney function. Furthermore, the extract has shown improvement on the histopathological changes of the pancreas, liver, and kidney in diabetes [27]. In another study, Kumar et al. reported the antihyperlipidemic activity of *Dillenia indica* (methanolic leaf extract) in Wistar rats. Diabetes was induced by a single intraperitoneal injection of STZ at a dose of 60 mg/kg body weight to induce diabetes. The animal was treated with the extract at two different doses 250 and 500 mg/kg p.o. for 21 days. The study found that the use of *Dillenia indica* could be beneficial in the management of diabetes and other abnormalities allied with this metabolic disorder [15].

### 3.3. *Dillenia indica* in Diabetes Associated Neuropathy

Diabetic neuropathy is a neurological disorder associated with diabetes mellitus. Inflammation is the main culprit in diabetic neuropathy [33]. Diabetic peripheral neuropathy is a long-term associated complication of diabetes mellitus and results in morbidity. One-quarter of patients underwent this type of complication [34]. Symptoms associated with these complications are tingling, hyperalgesia, burning, and pain [35, 36]. A study reported by Kaur et al. isolated an active compound “chromane” which is tested preclinically in rats and this isolated active compound from *Dillenia indica* has shown the beneficial effect in diabetic neuropathy. In this study, diabetic neuropathy is induced in rats by intraperitoneal administration of STZ at 65 mg/kg dose. Neuropathy development was confirmed from marked hyperalgesia and allodynia and reduced motor nerve conduction velocity (MNCV) was associated with increased formation of AGEs and reactive oxygen species. Alcoholic extract of *Dillenia indicia* (DAE) at a dose (100, 200, and 400 mg/kg, p.o.) and chromane at dose 5 and 10 mg/kg, p.o. were administered orally for 30 days from the 60th day of STZ administration. The study found that DAE and chromane ameliorated hyperglycemia and diabetic neuropathic pain via modulation of oxidative-nitrosative stress and reduction in AGE formation in the diabetic rats [29].

### 3.4. *Dillenia indica* in Diabetes-Associated Nephropathy

Diabetic nephropathy is one of the severe renal complications associated with diabetes. Proteinuria and renal dysfunction are the main clinical features of nephropathy [37]. Thickening of glomerular basement membrane and aggregation of mesangial matrix are the pathological features of diabetic nephropathy which leads to renal failure. The major cause of renal fibrosis and diabetic nephropathy are mesenchymal transdifferentiation of renal tubular epithelial cells [38]. Increased levels of advanced glycation end-products (AGEs) in the kidney of diabetes subjects might be responsible for all associated complications of diabetic nephropathy. Kaur et al. evaluated *Dillenia indica* for its in vitro inhibitory activity against the formation of AGEs by using bovine serum albumin. *Dillenia indica* showed significant inhibition of AGE formation in vitro. Diabetic nephropathy was induced in vivo by administration of a dose of STZ (65 mg/kg i.p.) 15 min after nicotinamide (230 mg/kg, i.p.). *Dillenia indica* extract at a dose of 100, 200, and 400 mg/kg showed nephroprotective effect in diabetic rats. *Dillenia indica* produced significant attenuation of glycemic status, renal parameter, lipid profile, and augmentation of antioxidant enzymes. All the effects together proving a protective effect in diabetic nephropathy. Moreover, *Dillenia indica* produced a significant reduction in the formation of AGEs in the kidneys. This study concluded that *Dillenia indica* might be a potential therapeutic agent for diabetic nephropathy [28].

## 4. Therapeutic Potential of *Dillenia indica* in Diabetes: Human Study

Human study with *Dillenia indica* conducted in Assam, India, at the Government Medical College and Hospital by Das and Sarma. The fruit powder of *Dillenia indica* has shown significant hypoglycemic effects in type 2 diabetes patients. In this study, 40 patients (nineteen male and twenty-one female) with type 2 diabetes were selected randomly but fulfilling all the inclusion criteria. They received fruit powder of *Dillenia indica* at dose 30 g/day (divided into two doses) half an hour before lunch and dinner with warm water for 24 weeks along with diet control and lifestyle modification. They are followed up at the 8^th^, 16^th^, and 24^th^ week. Both fasting and postprandial blood glucose levels of diabetic patients were reduced significantly without any adverse effect. This study concluded that the *Dillenia indica* fruit has a potential therapeutic value to treat hyperglycemia in humans [39].

## 5. *Dillenia indica* as Antidiabetic: Molecular Mechanisms

### 5.1. *Dillenia indica* as Alpha-Amylase and Alpha-Glucosidase Inhibitor

Alpha-amylase and alpha-glucosidase are two enzymes that digest carbohydrates in the gastrointestinal tract. Alpha-amylase causes hydrolysis of *α*-linked polysaccharides to make oligosaccharides, while alpha-glucosidases catalyzes the carbohydrates to monosaccharides that include glucose. The inhibition of these two enzymes reduces the postprandial rise in blood glucose levels [40]. Thus, alpha-amylase and alpha-glucosidase inhibitors reduce the deliverance of monosaccharides from dietary carbohydrates and decline in glucose absorption in blood stream, thereby averting a sudden increase in the blood glucose level after a meal [41, 42]. Kumar et al. reported that an isolated compound from *Dillenia indica* showed antidiabetic effect via inhibition of these two enzymes. In this study, seven active compounds were isolated. These are betulinic acid, n-heptacosan-7-one, n-nonatriacontan-18-one, quercetin, *β* sitosterol, stigmasterol, and stigmasteryl palmitate. These isolated compounds were administered to streptozotocin-nicotinamide-induced diabetic mice individually at 10 mg/kg dose. A single dose of the isolated compound or vehicle was administered orally by gastric intubation and the effect of these compounds on blood glucose level was determined in the animals at 0, 4, 8, and 24 h and compared with a standard drug. The data confirmed that out of seven compounds only four have shown alpha-amylase and alpha-glucosidase inhibitory activity. The study concluded that the antidiabetic activity of this plant extract work through inhibition of alpha-amylase and alpha-glucosidase activity [30].

### 5.2. *Dillenia indica* as AGE Inhibitors and Antioxidants

Diabetic microvascular complications, i.e., retinopathy, nephropathy, and neuropathy, are caused due to long-term hyperglycemia [43, 44]. Hyperglycemia causes an increase in glucose flux and other sugars through the polyol pathway, thereby increasing advanced glycation end-products (AGEs) intracellularly [45]. Evidence from various studies also indicated that hyperglycemia and elevated levels of AGEs may enhance reactive oxygen species (ROS) and oxidative stress [46–48]. Alcohol and hydroalcohol extract of *Dillenia indica* was evaluated for its in vitro inhibitory activity against AGE formation by using bovine serum albumin. *Dillenia indica extracts* showed significant inhibition of AGE formation in vitro [28]. Similarly, different doses of *Dillenia indica* extract (100, 200, and 400 mg/kg) were found to be effective to reduce renal AGE formation and oxidative stress in diabetic rats [28]. Plabita et al. also evaluated the role of *Dillenia indica* in diabetes-induced oxidative stress. The methanolic fruit extract (MEF) of *Dillenia indica* was administered to mice at a dose range of 150–550 mg/kg b.w. The study found that 350 mg/kg dose of MFE was found to be effective in alleviating the blood glucose level as well as oxidative stress [48]. Another study also showed that alcoholic extract of *Dillenia indica* (DAE) and isolated compound, chromane, showed high scavenging activity towards hydroxyl radical and superoxide anion, along with high reducing power activity [26]. The antioxidant effect of *Dillenia indica* could be considered to have a therapeutic value against diabetic complications.

## 6. Conclusion

**Figure 2:**
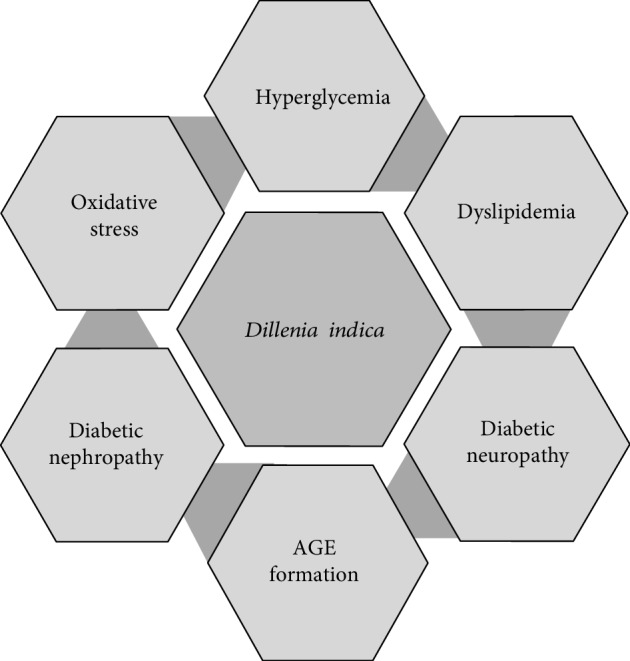
Therapeutic effects of *Dillenia indica* in diabetes and its associated complications.

Diabetes is considered one of the serious health concerns worldwide. There are various medicines and therapies available based on the principle of modern medicines but most of them show several side effects. Therefore, we need medicines having fewer side effects, cost-effective, and easily accessible to all. Diabetes and its complication treatment with herbal medicines have always a greater benefit as herb contains several hundred compounds and thus the possibility to work through multiple targets. In this review article, an effort has been made to cover the use of *Dillenia indica* in diabetes and its associated complications, and the various mechanisms by which this plant shows its beneficial effects (Figure 2). Scientific literature has proved the belief of using traditional herb, *Dillenia indica*, in the treatment of diabetes and its associated complications. *Dillenia indica* has promising effects in treating foremost aspects of diabetes including hyperglycemia and hyperlipidemia. In addition to this, it could be beneficial in the treatment of diabetes-associated complications including diabetic neuropathy and diabetic nephropathy. Regardless of these beneficial effects, detailed molecular studies are needed to be done in the future. Although previous literature looked for few mechanisms of the plant extracts for a beneficial effect in diabetes, researchers need to explore several other potential mechanisms, which include AMPK activation, AKT phosphorylation, GLUT4 translocation, insulin secretion from the pancreas [49], and mitochondrial respiration [50]. There is only one clinical study till now from Assam, Northeast region of India, where the antidiabetic potential of the plant fruit has been shown. Further, well-designed clinical studies are required to confirm the potential role of *Dillenia indica* in diabetes. An extensive investigation should also be carried out to isolate active compounds from *Dillenia indica* to develop small molecules for diabetic treatment.

## Figures and Tables

**Table 1:** Other names of *Dillenia indica*.

**Language**	**Vernacular names**

English	Elephant apple
Hindi	Chalta
Sanskrit	Avartaki
Assamese	Outenga
Bengali	Chalta
Sanskrit	Bhavya
Gujrat	Karambel

**Table 2:** Scientific classification.

Kingdome	Plantae
Super division	Spermatophyta
Division	Phanerogamae
Subdivision	Angiosperm
Class	Magnoliopsida
Subclass	*Dilleniidae*
Order	*Dilleniales*
Family	*Dilleniaceae*
Genus	*Dillenia* L.-*dillenia*
Species	*Dillenia indica* L.–chulta

**Table 3:** List of studies with *Dillenia indica* showing beneficial role in diabetes and its associated complications.

S. no.	Part used	Extract used	Phytochemical constituents	Dose	Animal/human study	Treatment	Reference
1.	Leaves	Methanolic extract	Not reported	(250 and 500 mg/kg b.w, p.o.)	Rat	Diabetes	[22]
2.	Leaves	Methanolic extract	Not reported	(250 and 500 mg/kg b.w, p.o.)	Rat	Hyperlipidemia in diabetes	[10]
3.	Leaves	Methanolic extract	Quercetin, *β* sitosterol, stigmasterol	(10 mg/kg b.w, p.o.)	Rat	Diabetes	[25]
4.	Leaves	Alcoholic extract (DAE)	Flavanoids, terpenoids, chromane	DAE (100, 200, and 400 mg/kg b.w, p.o.) and chromane (5 and 10 mg/kg b.w, p.o.)	Rat	Diabetes	[21]
5.	Leaves	Alcoholic extract (DAE)	Flavanoids, terpenoids, chromane	DAE (100, 200, and 400 mg/kg b.w, p.o.) and chromane(5 and 10 mg/kg b.w, p.o.)	Rat	Diabetic neuropathy	[24]
6.	Leaves	Alcoholic extract (DAE)	Terpenoids-betulinic acid	(100, 200, and 400 mg/kg b.w, p.o.)	Rat	Diabetic nephropathy	[23]
7.	Fruit	Aqueous and methanolic extract	Alkaloids, flavonoids, glycosides, tannins, terpenes, and saponins	(150–550 mg/kg b.w. p.o.)	Mice	Antihyperglycemic and antioxidant activity	[45]
8.	Fruit	Dried fruit powder	Tannins and reducing sugar	30 g/day	Human	Hypoglycemic activity	[35]
